# Heritable Variation in Courtship Patterns in *Drosophila melanogaster*

**DOI:** 10.1534/g3.114.014811

**Published:** 2015-02-03

**Authors:** Bryn E. Gaertner, Elizabeth A. Ruedi, Lenovia J. McCoy, Jamie M. Moore, Mariana F. Wolfner, Trudy F. C. Mackay

**Affiliations:** * Department of Biological Sciences, W. M. Keck Center for Behavioral Biology and Program in Genetics, North Carolina State University, Raleigh, North Carolina 27695-7614; † Department of Molecular Biology and Genetics, Cornell University, Ithaca, New York 14853-2703

**Keywords:** ethogram, quantitative genetics, behavior, DGRP, GWAS

## Abstract

Little is known about the genetic basis of naturally occurring variation for sexually selected behavioral traits. *Drosophila melanogaster*, with its rich repertoire of courtship behavior and genomic and genetic resources, is an excellent model organism for addressing this question. We assayed a genetically diverse panel of lines with full genome sequences, the *Drosophila* Genetic Reference Panel, to assess the heritability of variation in courtship behavior and mating progression. We subsequently used these data to quantify natural variation in transition probabilities between courtship behaviors. We found heritable variation along the expected trajectory for courtship behaviors, including the tendency to initiate courtship and rate of progression through courtship, suggesting a genetic basis to male modulation of courtship behavior based on feedback from unrelated, outbred, and genetically identical females. We assessed the genetic basis of variation of the transition with the greatest heritability—from copulation to no engagement with the female—and identified variants in *Serrate* and *Furin 1* as well as many other polymorphisms on the chromosome *3R* associated with this transition. Our findings suggest that courtship is a highly dynamic behavior with both social and genetic inputs, and that males may play an important role in courtship initiation and duration.

Multiple signals exchanged between potential mates during courtship may contribute to species and sex recognition and may provide a message of overall quality to the female (Candolin 2003). Courtship behavior may vary in the types of displays, or in the patterning of displays, depending on female preference. To place elaborate courtship into the context of continuously evolving sexually selected traits, one must first confirm that there is genetic variation for the *pattern* of behavior (or for the propensity to perform a specific behavior) within a species. It is also crucial to examine the precise genetic underpinnings of that behavioral variation. Gathering this information is necessary before a more thorough assessment of the role of sexual selection can begin (Kotiaho *et al.* 2008).

An ideal system for the exploration of elaborate behavioral patterns will include a documented genetic basis for complex behaviors that are well understood and well characterized (Hine *et al.* 2004). Mating behavior in the male fruit fly, *Drosophila melanogaster*, is composed of a complex series of discrete courtship behaviors, including orienting toward the female, following/chasing the female, tapping the female, performing wing vibrations that produce a species-specific courtship song, licking the female’s genitalia, attempting copulation by curving the abdomen toward the female, and finally copulation (Bastock and Manning 1955). Visual, auditory, and chemosensory cues between the male and female are an integral part of successful courtship (Markow 1987). Each behavior plays an important role in contributing to species recognition or assessment of the sex and receptivity of potential partners (reviewed in Greenspan and Ferveur 2000), and expressing all of these components in the appropriate situation and sequence increases the likelihood of mating success. Specific behaviors in this pattern are involved in reproductive isolation and are presumably the targets of sexual selection, including courtship song (Gleason and Ritchie 2004), as well as latencies and durations of courtship and copulation in response to species-specific pheromonal differences (Civetta and Cantor 2003).

The genetic and neural basis for the core components of male courtship behavior in *D. melanogaster* have been well documented (Messiner *et al.* 2011; Yamamoto and Koganezawa 2013) and involve the development of a sex-specific neural circuit via the genetic sex-determination cascade including the *fruitless* (Ryner *et al.* 1996) and *doublesex* genes (Taylor *et al.* 1994). Thus, natural variation in this behavior can, in principle, be tracked to its genetic and neuronal bases.

The sequence of male *D. melanogaster* behaviors culminating in successful courtship described previously typically is followed. However, deviations from the canonical progression of male mating behaviors (MMP, male mating progression) can be plastic in response to female behaviors and other cues (Siegel and Hall 1979; McRobert and Tompkins 1983). Further, there is evidence for genetic variation in the progression of male mating behaviors in naturally derived populations of *D. melanogaster* (Ruedi and Hughes 2008), *i.e.*, variation among lines in the durations and sequence of each courtship behavior. However, heritable variation in progression of behavior and transitions between behavioral states has yet to be examined. Shedding light on this could provide a more complete picture of the target for sexual selection and other selective pressures, because the *progression* of male courtship, and not just the discrete steps along the way, is under neural and genetic control (Markow 1987; Yamamoto and Koganezawa 2013).

Here, we use the naturally derived *D. melanogaster* Genetic Reference Panel (DGRP) of inbred lines with complete genome sequences (Mackay *et al.* 2012; Huang *et al.* 2014) to examine genetic variation and heritabilities for transitions between behavioral states. We evaluate whether there are extant behavioral “classes” in the form of different courtship patterns, including the propensity to transition from active courtship to attempted or full copulation. We further examine whether these classes are genetically variable, and we identify specific genetic underpinnings of the variation in these behavioral classes. The results presented here provide a framework for future research on sexual selection on behavioral patterns.

## Materials and Methods

### Animal husbandry

All experiments were performed using DGRP males (Mackay *et al.* 2012). The DGRP was derived from isofemale lines collected from the Raleigh Farmer’s Market (Raleigh, NC), whose progeny were inbred by full sib mating for 20 generations and subsequently maintained in small mass mating cultures under standard laboratory conditions (cornmeal-agar-molasses medium, 25°, 12-hr light-dark cycle). These lines have been fully sequenced and genotyped for single-nucleotide polymorphisms (SNPs), insertion-deletion polymorphisms, and karyotypes of common segregating inversions (Huang *et al.* 2014).

### Experimental flies

Virgin males were collected from bottles in which 10 males and 10 females were allowed to interact and reproduce for 2 d. For a few notoriously “weak” DGRP lines, bottles were populated with 20 males and 20 females to produce adequate numbers of virgins for assays. Females used in courtship assays were all F1 hybrids between two unrelated non-DGRP inbred strains, Oregon and Samarkand (Or × Sam). Thus, all females were genetically identical, yet genetically heterogeneous and robust. Virgin DGRP males and virgin Or × Sam target females were aged 3−5 d before behavioral assays in groups of 10.

### MMP assay

**Figure 1:**
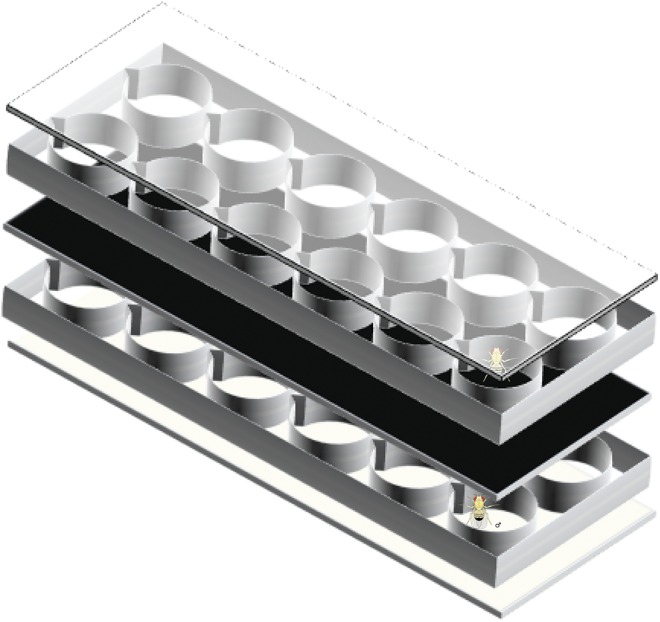
Copulatron schematic (expanded view). The copulatron is constructed of five layers: a CO_2_ pad (bottom), the lower chamber for *D. melanogaster* Genetic Reference Panel males, a thick opaque divider, the upper layer for tester (Or × Sam) females, and a clear lid. For simplicity, alignment posts, food, and a CO_2_ hookup are not shown. The divider is pulled out from between the two chambers to initiate the male mating progression assay. Copulatrons are modeled after Drapeau and Long (2000).

MMP (Ruedi and Hughes 2008) was observed using 8-arena “copulatrons” modeled after Drapeau and Long (2000) from Spring 2010 to Spring 2011 in five blocks (Figure 1). A total of 166 DGRP lines were assayed. Each line was assigned randomly to a block, and 10−15 males per DGRP line were assayed. Within each block, males were assigned randomly to trials and arenas of the copulatron to minimize the environmental variance; thus, multiple lines were assessed multiple days in a randomized manner. Approximately 18 hr before the start of the trial, males were lightly anesthetized with CO_2_ and placed in the lower portion of an arena. An opaque sheet of plastic was added, and virgin females (also lightly anesthetized with CO_2_) were placed in the upper portion of the arena. A clear Plexiglas lid was placed on the arena, and the flies were allowed to recover from anesthesia overnight at 25°. Both arena compartments contained fly food media to prevent starvation, desiccation, and general stress.

Pairs were observed in a 2-hr window beginning 1 hr after lights-on; eight pairs were assessed during a trial. At the beginning of the assay, the opaque divider was removed, exposing a single male to a single virgin female in an arena space of 2.5 cm diameter and 2.5 cm depth. The behavior of each male in each arena was recorded by scan sampling every 30 sec for 15 min, for a total of 30 observations per male. Behavior was recorded as one of eight ordinally scored options on the basis of previously described courtship behaviors (Ruedi and Hughes 2008). If a quick scan of the mating pair revealed the male transitioning between behaviors, the behavior closest to “copulation” on the MMP scale was recorded. By summing the score at each time point, any individual could have an overall MMP score ranging from 30 (not moving for the duration of the assay) to 240 (copulating for the duration of the trial). All observations were recorded by E.A.R. Trials in which either the male or female appeared injured or dead were not analyzed. After each trial, flies were discarded, and arenas were cleaned with soap, deionized water, and a small amount of bleach.

### Copulation duration

In a separate assay, copulation duration was assayed and compared with MMP assays for 35 DGRP lines chosen from the “core set” of 40 DGRP lines with the first broadly available sequence data. This assay was performed in two replicates, with at least 10 pairs per line assayed. Multiple strains (usually 3−5) were tested in a given experiment, and 50−75 vials were monitored concurrently in each experiment. Matings were set up in the morning, in glass vials containing a small piece of moistened filter paper. One unanesthetized 3- to 5-d-old virgin female was aspirated into each vial, followed by a 3- to 5-d-old unanesthetized male of the same DGRP genotype was added. Flies were observed continuously, and copulation duration, once initiated, was recorded for these lines. Copulations lasting less than 5 min were not included. All observations were recorded by J.M.M. Line means were calculated and compared with relevant courtship phenotypes. Flies with wing deformities or any other visible impairment were not used.

### Statistical analyses

All statistical analyses were performed using JMP 10.0 (SAS Institute, Cary, NC). Unless otherwise stated, dependent variables met the assumptions of general linear models. For analysis of noninitiating males, log likelihood and Akaike information criterion scores were used to determine the best-fitting distribution of observed data. Because noninitiating males were a line-dependent phenomenon, we chose to include these males in the subsequent analyses. For analysis of the frequency of specific courtship behaviors as well as overall mating success, line means and heritabilities were calculated with line as a random effect after taking block effects into account. Both raw and arcsin transformed data were analyzed, with no difference in the interpretation of the data. We applied a Bonferroni correction to account for multiple testing. For α = 0.05, the critical *P* value was 0.002. For α = 0.01, the critical *P* value was 0.0001.

### Edge-weighted ethogram construction

Edge-weighted ethograms can be used to quantify behavioral patterns from temporal data (Altmann 1974; Markow 1987; Chen *et al.* 2002), where connections (edges) between observed behavioral states are weighted by the frequency of transitions between these behavioral states. To create these ethograms from observed behaviors collected via scan sampling (*i.e.*, noncontinuous observations), we combined some behaviors into broader categories: “No movement” and “movement not engaging with female” were combined into a “no engagement with female” category. “Orienting towards female” and “approaching female” were combined into an “engagement with female” category, because these two behaviors may be conflated with scan sampling. “Wing vibration” and “licking genitalia” were considered independent categories. “Attempted copulation” and “copulation” were combined, because the purpose of this experiment is to examine male courtship progression and not female receptiveness.

Ethograms derived from scan sampling provide a lower boundary for transitions between behavioral states (Altmann 1974), because the experimental design causes us to miss events that last less than 30 sec. However, because courtship in *Drosophila* proceeds in a stepwise fashion (Bastock and Manning 1955; Greenspan and Ferveur 2000), ethograms constructed in this way still will depict accurately the progression through these stages as well as any large-scale deviations from the typical step-wise ordering (Collins *et al.* 1985).

Using a first-order Markov chain, we constructed transition matrices between these categories by calculating the frequency of a behavioral category at time *t+1* given the behavioral category observed at time *t*. Transition probabilities were calculated on an individual basis by dividing the number of observed transitions by the total number of transitions, such that the sum of all probabilities in a transition matrix for any individual is 1. Line means and heritabilities were calculated after taking block effects into account, treating line as a random effect.

### Principal components and factor analyses (FAs)

To test whether there was an overall pattern among transitions between behaviors, we performed a principal components analysis on the means of the 25 transition probabilities derived from the ethogram formation for each line. The first seven principal components had an eigenvalue of more than 1. These seven components were used in a FA using varimax rotation, and principal components were used to assign prior communality and as the factoring method.

### Genome-wide association study (GWAS)

We performed a GWAS on the transition probability with the greatest heritability—transitioning from copulation to no engagement with female (E to A)—to identify candidate genes. The GWAS was performed using PLINK (Purcell *et al.* 2007) by regressing line means of this transition probability on each of approximately 2,400,000 SNPs after correcting for cryptic relatedness, presence of inversions, and presence of *Wolbachia* (Huang *et al.* 2014). We assessed distribution of *P* values using a QQ plot and used a *P* value of 2.08 × 10^−8^ for a significance threshold (α = 0.05) after a strict Bonferroni correction.

## Results

### Heritable variation for courtship behaviors

To test whether there was natural genetic variation for specific steps in courtship, we assayed DGRP males against F1 females derived from a cross of two inbred lines not related to the DGRP by scan sampling behaviors (Table 1 and Supporting Information, File S1) over a period of 15 min. We found that most recorded behaviors showed modest but significant broad sense heritability after accounting for multiple tests (*P* < 0.0001 for an experimentwise *α* = 0.01), ranging from *H*^2^ = 0.033 (approach female) to *H*^2^ = 0.094 (copulation) (Table 2). These behaviors subsequently were condensed into broader categories to account for possible ambiguity in interpreting behavioral transitions because the data were gathered via scan sampling as opposed to continuous observation (Table 1). Using this new classification, we found that the greatest heritability was *H*^2^ = 0.11 (E, male initiates / succeeds in copulation) whereas the lowest was *H*^2^ = 0.060 (D, genital licking).

**Table 1:** Ordinal phenotypes for male mating progression

Description	Original Score	Condensed Score	Description
Male is not moving and not in close proximity to female	1	A	Male not engaged with female
Male is moving but not engaging with female	2		
Male is orienting in a semicircle around female	3	B	Male is in close proximity to female, is pursuing her
Male is pursuing female	4		
Male has wing extended at a 90° angle in rapid vibration	5	C	Male has wing extended at a 90° angle in rapid vibration
Male is positioned behind female and proboscis is on her genitals	6	D	Male is positioned behind female and proboscis is on her genitals
Male is behind female with abdomen in a “c” curve to initiate copulation	7	E	Male attempts or has succeeded in copulating
Male and female are copulating	8		

**Table 2:** Analysis of variance of male mating phenotypes

Trait	% B	FB	PB	H2, %	FL	PL
MMP (summary)	3.30	12.24	<0.0001*	9.04	2.03	<0.0001*
1) no movement	0.24	1.79	0.126	9.09	1.67	<0.0001*
2) movement	5.85	21.44	<0.0001*	8.37	1.95	<0.0001*
3) orient toward female	1.84	7.18	<0.0001*	5.02	1.55	<0.0001*
4) approach female	2.73	10.23	<0.0001*	3.32	1.36	0.0028
5) wing vibration	0.82	3.72	0.0051	9.23	2.06	<0.0001*
6) genital lick	0.37	2.23	0.0639	5.59	1.17	<0.0001*
7) attempted copulation	0.74	3.45	0.0081	2.92	1.13	0.0069
8) copulation	2.33	8.85	<0.0001*	9.39	2.08	<0.0001*
A (no movement and movement)	3.81	14.04	<0.0001*	11.21	2.31	<0.0001*
B (orient and approach)	3.76	13.84	<0.0001*	9.14	2.05	<0.0001*
C (wing vibration)	0.82	3.72	0.0051	10.01	2.16	<0.0001*
D (genital licking)	0.37	2.23	0.064	6.02	1.67	<0.0001*
E (attempt and copulate)	2.55	9.62	<0.0001*	11.31	2.23	<0.0001*

% B is the % of the total variation attributable to the effect of Block. H2 is the broad sense heritability (after accounting for the block effect). FB and FL are, respectively, the F ratio statistics for the Block and Line effects, and PB and PL are the corresponding *P* values.

* Indicates significance at α = 0.01 after a Bonferroni correction across all statistical tests performed on all phenotypes (nominal *P* = 0.0001)

### A subset of lines is less likely to initiate courtship

**Figure 2:**
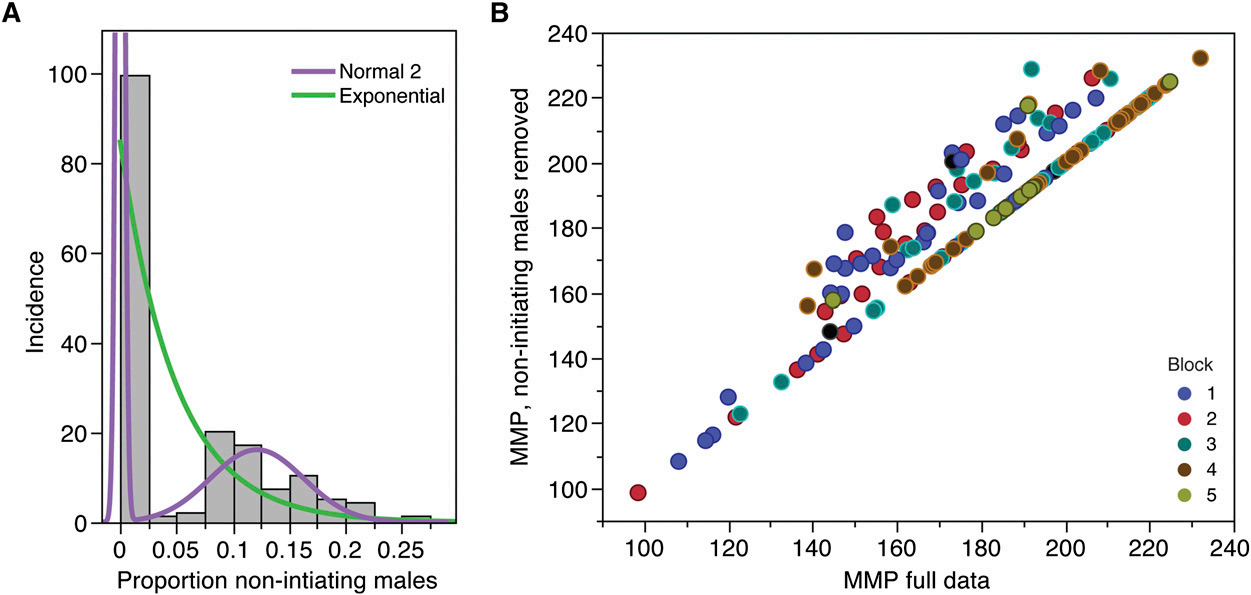
Distribution of noninitiating males is line-dependent. The percentage of males that never initiate courtship was quantified by line. (A) The distribution of these males fits a normal 2 mixture model (purple line) better than an exponential model (green line), where most lines have zero noninitiating males (µ1 = 4.96 × 10−6), and some lines have a modest proportion of noninitiating males (µ2 = 0.122). (B) Noninitiating males are found exclusively among lines with mid- to high-range male mating progression scores, calculated by comparing average line MMP scores with noninitiating males removed *vs.* included in the analysis. Blocks are color coded.

A subset of males never initiated any interaction with females during the courtship assays, failing to score greater than “2” (movement) on the MMP scale at any point of observation over the duration of the assay. To explore this further, we analyzed the incidence of noninitiating males by line (Figure 2A). We fit distributions to the data and found that a Normal 2 mixture model fits better than an exponential model (Akaike information criterion = −950.75 and −666.56, respectively). The presence of two normal curves suggests that there are two distinct groups that differ in initiation. That is, in most lines, all males initiated courtship with females, but in a few lines roughly 15% of males never initiated. We then compared the overall MMP score for these lines including or excluding the noninitiating males (Figure 2B). Somewhat surprisingly, we found that noninitiating males are found exclusively in lines with a moderate- to high-MMP score (140−210), but are almost entirely absent in lines with low MMP scores (under 140). To test whether these lines are generally impaired for locomotion, we correlated the proportion of males not initiating per line with previously assayed startle response data (Jordan *et al.* 2007). We found no relationship between the two (Figure S1). Thus, it is not the case that low MMP lines often do not initiate courtship, nor is it the case that noninitiating males are physically less able to court. Instead, males from moderate MMP lines may possess a threshold to initiate courtship, and once that threshold is passed, they are typically successful at copulating, whereas males from low MMP lines always initiate courtship behavior but often are not successful at copulating within the timeframe of this assay.

### Heritable variation in edge-weighted ethograms

The decision to initiate courtship behaviors appears to be line dependent. Therefore, we next tested whether there was also variation in the sequence of the broader courtship behavior categories. We constructed ethograms to describe the courtship behavior of each individual and tested the extent to which variation in those ethograms was genetically based. Overall, we found that pairs were most likely to be in copulation (*P*(E to E), 0.61) or not interacting (*P*(A to A), 0.18) between two sequential observations. Many transitions occurred with equal probability across all possible categories, which enforces the important role of female interaction, because her rejection of the male would cause these shifts in behaviors regardless of male genetic background (Markow 1987).

**Figure 3:**
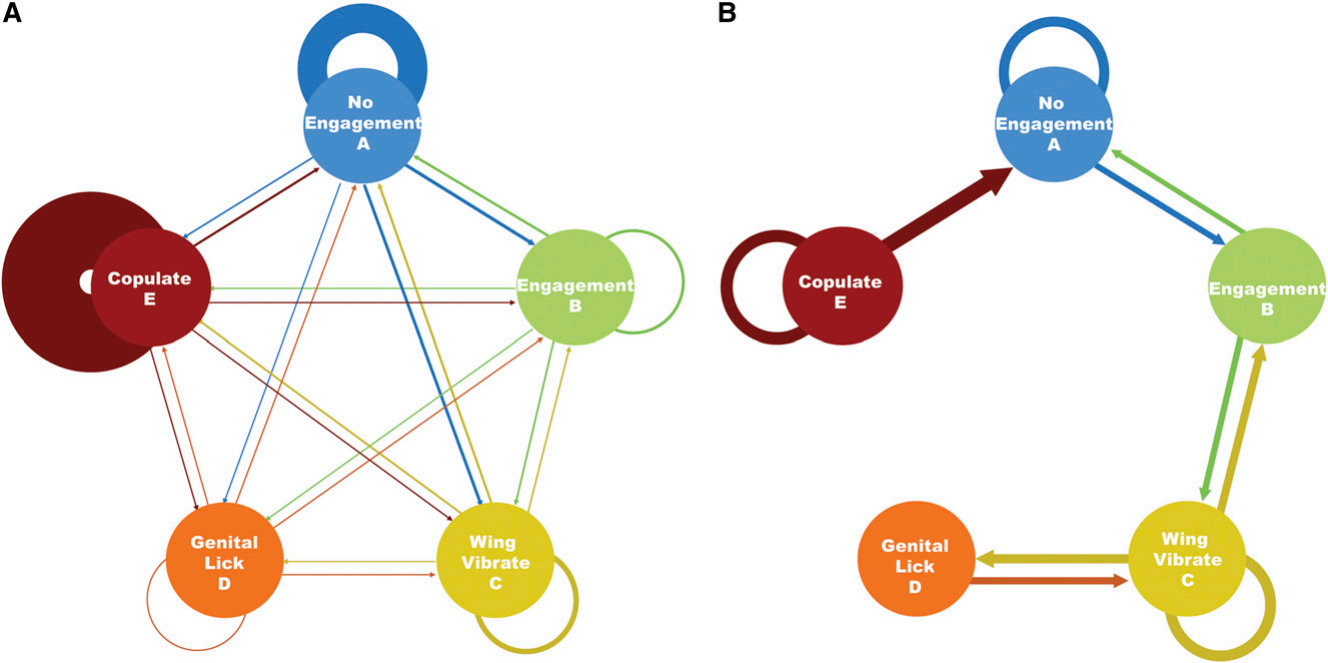
Transitions between courtship behaviors vary in frequency and are heritable. Reduced courtship behaviors are arranged according to previously-reported increase in courtship intensity from A to E. A: no engagement with female; B: orienting or approaching female; C: wing vibration; D: genital licking of female; E: attempted or successful copulation. (A) Ethogram describing transition probabilities between each state for transitions averaged across all *D. melanogaster* Genetic Reference Panel lines. The line width is proportional to the proportion of transitions observed. (B) Heritable transitions. The line width is proportional to the percent of genetic variation for each transition probability. Only genetically variable (*P* < 0.002, accounting for multiple tests with an experimentwise α = 0.05) transitions are indicated.

To test whether male genetic background influenced mating progression patterns, we performed an analysis of variance on each transition probability with DGRP line as a main effect. Overall, continuous behavioral states (*i.e.*, A to A; B to B; etc.) exhibited some of the greatest heritable variation (Table 3), along with relatively high levels of heritable variation for transitions in courtship behaviors expected for “stereotypical” courtship patterns (*i.e.*, A to B, B to C, C to D), (Figure 3B and Table 3). This finding supports the idea that males with different genetic backgrounds progress through courtship at different rates, either because the female is more receptive to different genetic backgrounds or because the males perceive or respond to female receptivity differentially due to their own genetic background. If true, then transition-probability variation should correspond with overall MMP scores.

**Table 3:** Heritable variation in transition probabilities

t	t+1	% B	FB	PB	H2	FL	PL	Mean
A	A	2.97	11.06	<0.0001*	7.80%	1.88	<0.0001*	0.23
A	B	6.39	23.47	<0.0001*	4.73%	1.52	<0.0001*	0.025
A	C	4.10	2.35	0.0517	−0.50%	0.95	0.6624	0.023
A	D	−0.14	0.53	0.7138	−0.72%	0.99	0.5134	0.0049
A	E	0.48	2.58	0.036	2.75%	1.29	0.0098	0.015
B	A	4.26	15.65	<0.0001*	3.79%	1.41	0.0009*	0.019
B	B	2.49	9.41	<0.0001*	3.17%	1.34	0.0039	0.022
B	C	0.29	1.96	0.0982	4.96%	1.54	<0.0001*	0.014
B	D	−0.06	0.80	0.5277	1.02%	1.11	0.1778	0.0034
B	E	2.20	8.41	<0.0001*	0.41%	1.04	0.3477	0.0093
C	A	0.09	4.10	0.003	−1.52%	0.885	0.9184	0.017
C	B	3.18	2.05	0.0852	6.27%	1.70	<0.0001*	0.012
C	C	1.44	5.79	<0.0001*	8.46%	1.96	<0.0001*	0.042
C	D	0.38	2.25	0.0617	7.17%	1.80	<0.0001*	0.087
C	E	0.63	3.08	0.0154	1.68%	1.18	0.0706	0.015
D	A	1.41	1.46	0.2105	0.20%	1.00	0.04868	0.0039
D	B	−0.07	0.77	0.5461	1.69%	1.18	0.0694	0.0029
D	C	0.51	2.67	0.0308	5.63%	1.62	<0.0001*	0.0080
D	D	0.56	2.86	0.0225	1.28%	1.14	0.1272	0.0050
D	E	−0.21	0.301	0.8775	1.55%	1.16	0.0862	0.0030
E	A	1.46	5.88	0.0001*	11.80%	2.39	<0.0001*	0.017
E	B	0.90	3.97	0.0033	3.06%	1.33	0.005	0.0023
E	C	−0.11	0.66	0.6234	1.35%	1.14	0.116	0.0043
E	D	0.06	1.19	0.3122	0.24%	1.03	0.404	0.0020
E	E	2.40	9.09	<0.0001*	9.51%	2.09	<0.0001*	0.69

t and t+1 indicate the behaviors in successive observation periods. % B is the % of the total variation attributable to the effect of Block. H2 is the broad sense heritability (after accounting for the block effect). FB and FL are, respectively, the F ratio statistics for the Block and Line effects, and PB and PL are the corresponding *P* values. Mean is the average transition probability across all DGRP lines.

* Indicates significance at α = 0.05 after a Bonferroni correction across all statistical tests performed on all phenotypes (nominal *P* = 0.002).

### Elements of courtship ethograms correspond with variation in MMP

**Figure 4:**
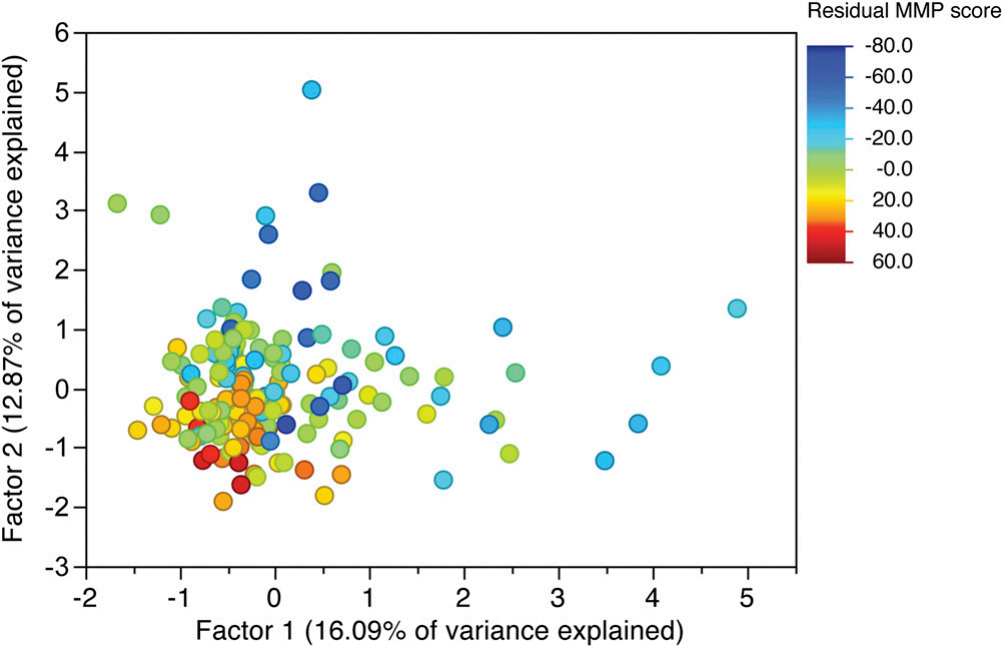
Factor analysis of ethogram data corroborates overall courtship behavior. Each dot represents Factor 1 and Factor 2 scores for a line and is colored according to the average residual male mating progression score of that line after accounting for block effects. Lines with high average MMP scores (warm colors) tend to show low variation in transition matrices. They are distinguished from lines with middle MMP scores (light blues) along Factor 1 and from lines with low MMP scores (dark blues) along Factor 2.

We identified heritable variation in the courtship patterns of male *D. melanogaster*. To test whether discrete differences in transition probabilities might have a multivariate covariance structure, we performed a FA on the line means of each behavioral transition probability based on the significant eigenvectors in a principal component analysis (see the section *Materials and Methods*). Factor 1 explained 16.09% of the variance, and Factor 2 explained 12.87% of the variance in the transition matrices (Table 4). Both Factor 1 and Factor 2 load negatively with a copulation−copulation transition, and overall loading weights are uncorrelated between Factor 1 and Factor 2 (*r* = 0.13, *P* = 0.51). We compared these factors with the overall MMP score (Figure 4) and found that lines with high probabilities for copulation−copulation transitions (loading negatively on Factors 1 and 2) had the greatest MMP scores, which is internally consistent as these males are observed copulating the most. Factor 1 distinguishes these high MMP males from those with mid-range MMP scores, whereas Factor 2 distinguishes high MMP males from those with the lowest MMP scores. Thus, although there is little variation among lines with high copulation success, it appears that lines with low and moderate success are performing qualitatively different behaviors.

**Table 4:** Factor loadings for multivariate analysis of behavioral patterns

t	t+1	F1 (16.09)	F2 (12.87)	F3 (12.35)	F4 (8.54)	F5 (8.43)	F6 (6.66)	F7 (6.33)
A	A	0.084	0.305	0.067	−0.206	−0.408	−0.105	0.680
A	B	0.050	0.875	0.126	0.114	−0.088	0.126	−0.021
A	C	0.106	0.226	0.780	0.008	0.234	−0.011	0.032
A	D	0.217	−0.173	0.644	0.263	−0.129	0.048	0.165
A	E	−0.289	−0.046	0.100	0.227	0.172	−0.217	0.435
B	A	−0.018	0.895	0.114	0.079	−0.011	−0.055	0.082
B	B	0.259	0.694	−0.109	−0.004	0.190	0.333	−0.001
B	C	0.591	0.538	0.102	−0.181	0.207	0.103	−0.141
B	D	0.615	0.173	−0.055	0.245	−0.061	0.365	−0.012
B	E	−0.004	0.092	0.066	0.002	−0.011	0.865	−0.136
C	A	0.042	0.240	0.824	−0.017	0.157	0.083	0.028
C	B	0.540	0.483	0.252	−0.202	0.136	0.141	−0.156
C	C	0.649	0.050	0.464	−0.205	0.327	−0.132	−0.155
C	D	0.784	−0.021	0.350	0.216	0.237	−0.104	−0.053
C	E	0.174	0.032	0.013	0.092	0.772	−0.007	−0.017
D	A	0.139	−0.010	0.695	0.365	−0.080	−0.002	0.130
D	B	0.673	0.172	−0.205	0.147	−0.006	0.286	0.173
D	C	0.787	−0.016	0.384	0.177	0.193	−0.125	−0.012
D	D	0.584	0.032	0.123	0.499	0.133	−0.087	0.095
D	E	0.138	−0.042	0.226	0.789	0.137	0.127	−0.100
E	A	−0.028	−0.195	0.102	0.147	0.315	0.136	0.634
E	B	0.098	0.192	0.053	0.010	0.426	0.609	0.197
E	C	0.152	0.032	0.163	0.109	0.757	0.120	0.103
E	D	0.087	0.119	0.064	0.788	0.081	−0.046	0.122
E	E	−0.473	−0.499	−0.361	0.094	0.078	0.022	−0.516

t and t+1 indicate the behaviors in successive observation periods. Numbers in parentheses are the % variance explained for each factor. Underlined numbers indicate strong contribution to factor weights. FN, factor number.

### Variation in highly heritable transition maps to a cluster of genes on *3R* and to other loci

**Figure 5:**
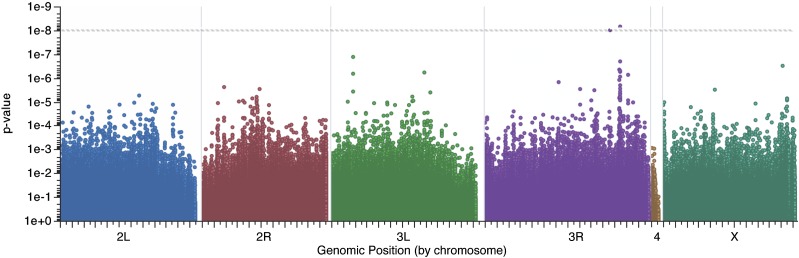
Genome-wide association study of transition probability. *P* values of associations of single-nucleotide neoplasms with the transition probability of “copulating” to “not engaging with female” (E to A) are plotted by genomic position. Two variants on chromosome 3R are significant following a Bonferroni correction for multiple tests (horizontal dashed line)

The transition with the greatest heritable variation was “copulation” to “no engagement” (E to A, Table 3). To test whether we could identify specific loci that correspond with this transition, we performed a GWAS on this phenotype (Figure 5). Two intronic SNPs in *Serrate* (*Ser*) and *Furin 1* (*Fur1*) passed a stringent Bonferroni correction (*P* = 6.97 × 10^−9^ and 9.89 × 10^−9^, respectively) for multiple tests, and 57 additional SNPs with *P* values ≤ 10^−5^ (Table S1) were associated with variation in this transition. In addition to the SNP in *Ser* with the strongest association, 18 of these top SNPs were located in *Ser* introns and regulatory elements.

### Transition probabilities predict copulation duration in an independent assay

**Figure 6:**
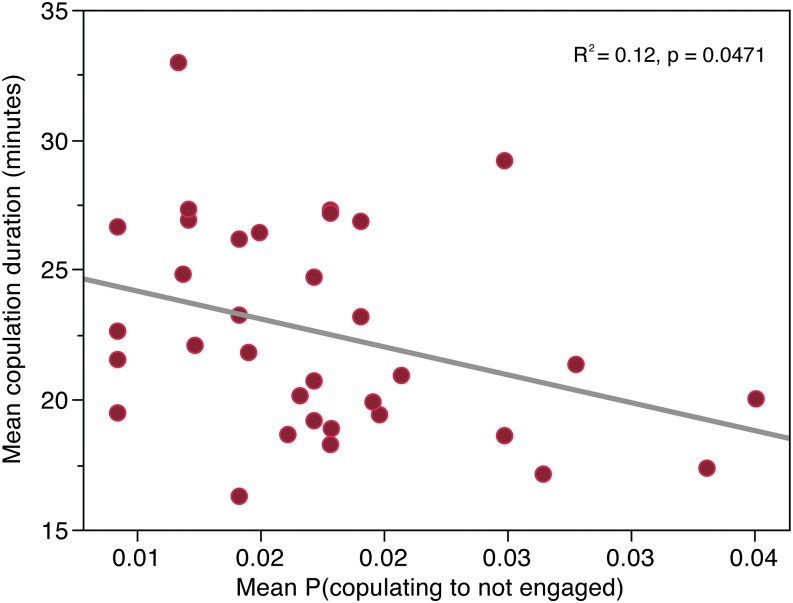
Ethogram transition probabilities predict other phenotypes. The line mean for probability of observing the “no engagement” behavioral state from the “copulating” state predicts duration of copulation in a subset of *D. melanogaster* Genetic Reference Panel lines.

Courtship behavior is genetically variable but also dependent on environment and receptiveness of the female. Thus, we anticipated a modest correlation between related behaviors across assays. To test that these ethograms are predictive, we performed an independent experiment on copulation duration using 35 DGRP lines. We found that copulation duration has a broad sense heritability of *H*^2^ = 0.33 (F_34,979_ = 15.38, *P* < 0.0001). This assay differed from the MMP assay in three ways. First, the male DGRP was paired with a female from the same line instead of an unrelated common tester female. Second, assays were performed in glass vials rather than a copulatron. And finally, these flies were not anesthetized 24 hr before the assay. Despite these experimental differences, we identified a slight but significant negative relationship (*r* = 0.35, *P* = 0.047) between copulation duration and the probability of transitioning from “copulation” to “no engagement” (E to A) in these 35 lines (Figure 6). This transition may reflect the extent to which males control copulation duration. Although correlated at the phenotypic level, there was no significant association between the previously mapped loci and the copulation duration phenotype or the reduced E to A phenotype, likely because the smaller sample size reduces the representation of causal variants in the GWAS.

## Discussion

By using a multifaceted analysis that combined observation of different courtship behaviors, quantification of courtship intensity, and calculation of transition probabilities between different behaviors, we were able to identify heritable variation in discrete courtship behaviors and overall courtship patterns. This type of analysis captures progress in male courtship behavior (*i.e.*, the sequence of behaviors and their relative timing) and demonstrates the extent to which there is genetic variation in that progress. We were able to quantify genetically based differences in the speed of courtship progression, the order of courtship behaviors, and rates of initiation of courtship behaviors and copulation. We further demonstrated that some behaviors are predictive of others in independent assays, indicating a robust genetic component.

These data were collected via scan sampling to maximize the number of individuals assessed while minimizing environmental heterogeneity that would have been introduced by doing fewer simultaneous trials. Because the data were not collected via a strict focal following method, any behaviors that lasted less than 30 sec were not recorded since they occurred between scans. Thus, this study provides only a rough approximation of male mating strategy. Ethograms and transitions constructed from data collected via scan sampling should be interpreted cautiously; for example, one of the first papers examining male behavior in *Drosophila* used scan sampling every 1.5 sec to account for the short duration of some behaviors (Bastock and Manning 1955). Despite this caveat, because behavior generally occurred along the ordinal direction described in Table 1 and because we were able to observe “quick” behaviors like licking, we can still draw inferences regarding courtship progression and patterns despite the time between observational periods. These data provide a framework for more rigorous assessment of MMP in the future.

Our data suggest that there are four different classes of genetically differentiated courting patterns in the DGRP. The first class consists of males from most DGRP lines, which readily initiate canonical *Drosophila* courtship behaviors and progress through to copulation and have the greatest MMP scores. The second class consists of lines with mid-range MMP scores with a high frequency of “noninitiating” males, which fail to initiate any type of courtship. Within these lines, though, males that *do* initiate courtship are usually successful at copulating. Our FA identified a third class of males which tend to initiate courtship without eventually achieving a successful copulation, or with delayed copulation (loading positively on factor 1). The fourth class consists of males that are less likely to progress in courtship at all, or are perhaps frequently rejected by the females (loading positively on factor 2). Interestingly, lines with non-initiating males tended to cluster with high MMP males in the FA (loading low on both factors 1 and 2), suggesting that any threshold effect is independent of the transition patterns that distinguish the slower maters from each other. The design of this study precludes analysis of any social factors that differentiate these two “slow” groups of males from each other, and assessing why some DGRP males have a greater threshold to initiate courtship behaviors. One possibility is that the common tester female genotype only permits mating by certain genotypes; this is consistent with female “choosiness” based on hydrocarbon profile (Gleason *et al.* 2005; Veltsos *et al.* 2012). Alternatively, these males may harbor genetic variation in elements of their courtship neural circuitry that affects their propensity to mate with this particular female genotype.

We performed a GWAS for the transition probability with the greatest heritability—the progression from “copulation” to “no engagement”—to identify candidate genes affecting this variation. The strongest signal in our GWAS was a SNP in *Serrate*, a Notch ligand (Fleming *et al.* 1990) that is essential for ectodermal development and cell differentiation. It has not been implicated previously in phenotypes related to male mating behavior, though some alleles (*Ser*^rev 5-5^, Fleming *et al.* 1990) cause lethally defective neurodevelopment. Possibly naturally segregating polymorphisms that alter the time or level of expression of this gene are changing the overall nervous system structure, including neurons that ultimately are involved in the circuitry for courtship. In particular, neural circuitry set up by Ser and further masculinized through the effects of Dsx^M^ and Fru^M^ may bias males of a certain genotype to terminate copulation earlier that males with a different genotype.

The second significant SNP is in linkage disequilibrium with the *Ser* SNPs (although it is not in close physical proximity to *Ser*) and is located in regulatory elements of the *Furin 1* gene. Although its role in behavior is unclear, it is a peptidase that is involved in synaptic target recognition (Kurusu *et al.* 2008), and tergite pigmentation (Mummery-Widmer *et al.* 2009). As the pigmentation pathway relies on neurotransmitter precursors (Wright 1987), both *Ser* and *Fur1* remain strong candidates for causally influencing the male decision to cease copulation

The significant association of this behavioral transition and a particular locus indicates that the “decision” to continue copulation (*vs.* decoupling and losing interest, or decoupling and continuing to court) is not solely based on the reception or “decision” of the female, but also depends on the male genotype. This finding is supported by our final experiment, where we found a significant correlation between the “copulation to no engagement” transition probability and copulation duration in an independent assay with a different female genotype.

In summary, we were able to identify heritable variation in courtship patterns and progression across genetically diverse lines by transforming courtship data into an ethogram that is weighted by transition probabilities. The core components of courtship are fairly canalized. However, genetic variation in courtship patterns provides the substrate for response to natural or sexual selection for courtship duration, copulation duration, and even the order of courtship behaviors.

## 

## Supplementary Material

Supporting Information
