# Supplementary material for: Heritable Variation in Courtship Patterns in Drosophila melanogaster
Source: G3 (Bethesda). 2015 Feb 3;5(4):531–9. doi: 10.1534/g3.114.014811 (PMC4390569; doi:10.1534/g3.114.014811)
Supplement: Supporting Information [file supp_5_4_531__index.html]

Heritable Variation in Courtship Patterns in Drosophila melanogaster — Supporting Information 

# Heritable Variation in Courtship Patterns in *Drosophila melanogaster*

## Supporting Information for Gaertner *et al.*, 2015

**Files in this Data Supplement:**

- Supporting Information - Figure S1, Table S1, and File S1 (PDF, 287 KB)
- Figure S1 - Low correlation between the proportion of "dud" males and a measure of general locomotor activity, startle response. (PDF, 110 KB)
- Table S1 - Top SNPs from the GWAS for E-A transition probability. (PDF, 129 KB)
- File S1 - Raw data used in the analyses. (.xlsx, 415 KB)
