# Supplementary material for: Heritable Variation in Courtship Patterns in Drosophila melanogaster
Source: G3 (Bethesda). 2015 Feb 3;5(4):531–9. doi: 10.1534/g3.114.014811 (PMC4390569; doi:10.1534/g3.114.014811)
Supplement: Supporting Information [file supp_g3.114.014811_014811SI.pdf]

G3 · Genes | Genomes | Genetics

Heritable Variation in Courtship Patterns in *Drosophila melanogaster*

Bryn E. Gaertner[*†1], Elizabeth A. Ruedi[*‡1], Lenovia J. McCoy[*], Jamie M. Moore[§**], Mariana F. Wolfner[§], Trudy F.C. Mackay[*]

* Department of Biological Sciences, W. M. Keck Center for Behavioral Biology and Program in Genetics, North Carolina State University, Raleigh NC
[§] Department of Molecular Biology and Genetics, Cornell University, Ithaca NY
[†] Present address: Prescott Medical Communications Group, Chicago IL.
[‡] Present address: Genetics Society of America, Bethesda MD
[**] Present address: Department of Neurology and Neuroscience, Cornell-Weill Medical College, New York NY.

[1] Equal Contributions

Corresponding author:
Trudy F. C. Mackay
Department of Biological Sciences
Campus Box 7614
North Carolina State University
Raleigh, NC 27695-7614
Tel: 919-515-5810
Fax: 919-515-5810
Email: trudy_mackay@ncsu.edu

DOI: 10.1534/g3.114.014811

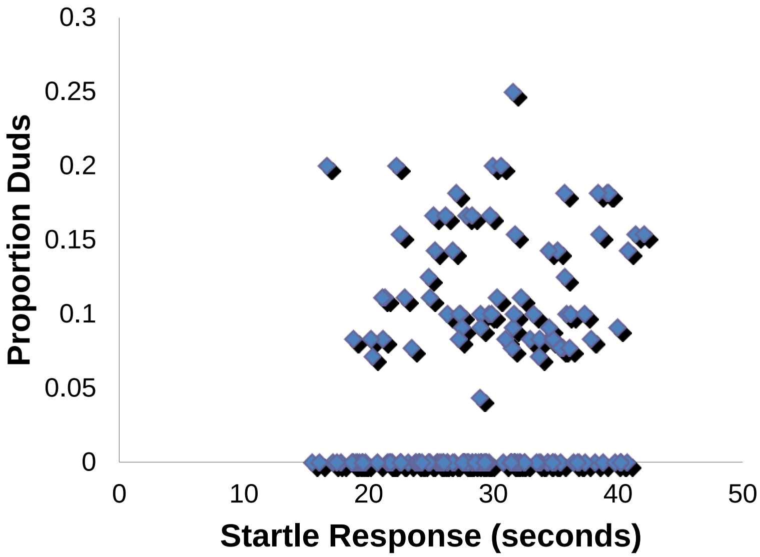

Figure S1. Low correlation between the proportion of 'dud' males and a measure of general locomotor activity, startle response. $r^2 = 0.0383$.

Table S1 Top SNPs from the GWAS for E-A transition probability. Location is noted by both chromosome and genomic location. The minor allele frequency (MAF) was obtained by dividing the number of lines with the frequent allele by the total number of lines tested at that locus. The effect is one-half the difference in mean between lines containing the major and minor allele (Falconer and Mackay, 1996). The *P*-value is from a mixed model analysis that takes *Wolbachia* infection status and relatedness due to shared inversion karyotypes as well as cryptic genetic relatedness into account. Probable gene as well as effect the polymorphism has on that gene is noted as derived from Flybase (Marygold *et al.* 2013). * indicates significant after Bonferroni correction ($\alpha = 0.05$, $P = 2.08 \times 10^{-8}$).

Genomic Location	MAF	Effect	*P*-value	Gene	Site Class
3R _23010872	0.075	-0.0062	6.97E-09*	*Ser*	Intron
*3R*_21296904	0.054	-0.0075	9.89E-09*	*Fur1*	Intron
*3L*_3706152	0.086	-0.0051	1.31E-07	Intergenic	N/A
*3R*_23009915	0.061	-0.0061	2.03E-07	*Ser*	Intron
*X*_20318587	0.129	-0.0044	3.10E-07	*CG42578*	Downstream
*3R*_22864199	0.0556	-0.0065	4.46E-07	*NepYr*	Intron
*3R*_23003275	0.067	-0.0056	5.04E-07	*Ser*	Synonymous
*3R*_22998908	0.073	-0.0056	5.54E-07	*Ser*	Synonymous
*3L*_15807977	0.058	-0.0067	5.92E-07	*fwe*	Intron
*3L*_3706141	0.097	-0.0047	6.65E-07	Intergenic	N/A
*3R*_24359687	0.068	-0.0057	7.41E-07	*Ppn*	Intron
*3R*_23004871	0.100	-0.0047	8.85E-07	*Ser*	Intron
*3R*_22864182	0.061	-0.0060	1.21E-06	*NepYr*	Intron
*3R*_12558223	0.150	-0.0042	1.50E-06	*Ubx*	Intron
*3R*_23006864	0.055	-0.0061	2.03E-06	*Ser*	Intron
*2R*_3790141	0.095	-0.0047	2.42E-06	*CG30377*	Intron
*3R*_23005060	0.067	-0.00523	2.79E-06	*Ser*	Intron
*2R*_9824749	0.200	-0.0035	2.93E-06	*shot*	Intron
*3R*_6210960	0.342	-0.0029	2.93E-06	*mun*	Intron
*X*_800458	0.085	-0.0053	3.12E-06	*e(r)*	Upstream
*3R*_8624797	0.490	-0.0026	3.29E-06	Intergenic	N/A

Genomic Location	MAF	Effect	*P*-value	Gene	Site Class
*3R*_3007818	0.059	-0.0058	3.70E-06	*Ser*	Intron
*3L*_706240	0.072	-0.0048	3.73E-06	Intergenic	N/A
*3L*_6821987	0.094	-0.0048	4.07E-06	*CG13024*	Start gained
*3R*_2999273	0.061	-0.0056	4.53E-06	*Ser*	Synonymous
*3R*_3001738	0.0549	-0.0057	4.86E-06	*Ser*	Intron
*2L*_3477359	0.237	-0.0033	5.49E-06	*CG42784*	Intron
*3L*_3766554	0.198	-0.0036	6.12E-06	*bru-3*	Intron
*3R*_2925879	0.063	-0.0059	6.20E-06	*eater*	Upstream
*3R*_3012273	0.069	-0.0055	6.21E-06	*Ser*	Intron
*2R*_312457	0.385	-0.0025	6.26E-06	*CG42321*	Intron
*3R*_2864293	0.158	-0.0039	7.03E-06	*NepYr*	Intron
*X*_1066955	0.115	-0.0038	7.24E-06	Intergenic	N/A
*2R*_586976	0.106	-0.0041	7.26E-06	*wuc*	Upstream
*3R*_2999769	0.067	-0.0053	7.46E-06	*Ser*	Synonymous
*X*_1067153	0.114	-0.0038	7.65E-06	Intergenic	N/A
*3R*_3002423	0.105	-0.0042	8.10E-06	*Ser*	Intron
*2R*_9337098	0.106	-0.0037	8.37E-06	*cnn*	Intron
*3R*_23009365	0.062	-0.0054	8.74E-06	*Ser*	Intron
*X*_21066980	0.110	-0.0039	8.75E-06	Intergenic	N/A
*3R*_24360681	0.055	-0.0058	8.77E-06	*Ppn*	Intron
*3R*_18106804	0.288	0.0030	8.79E-06	*SKIP*	Intron
*2R*_7000740	0.110	-0.0041	9.08E-06	Intergenic	N/A
*3L*_13938151	0.269	0.0027	9.10E-06	*CG32137*	Intron
*2R*_9312454	0.394	-0.0024	9.31E-06	*CG42321*	Intron
*3L*_13766585	0.195	-0.0035	9.49E-06	*bru-3*	Intron
*2R*_6301231	0.200	-0.0034	9.80E-06	*CG42732*	Intron
*3L*_2799032	0.354	0.0027	9.99E-06	*CG43444*	Intron

File S1

Raw data used in the analyses.

Available for download as an Excel file at http://www.g3journal.org/lookup/suppl/doi:10.1534/g3.114.014811/-/DC1
