# Supplementary material for: Heritable Variation in Courtship Patterns in Drosophila melanogaster
Source: G3 (Bethesda). 2015 Feb 3;5(4):531–9. doi: 10.1534/g3.114.014811 (PMC4390569; doi:10.1534/g3.114.014811)
Supplement: Supporting Information [file supp_g3.114.014811_FigureS1.pdf]

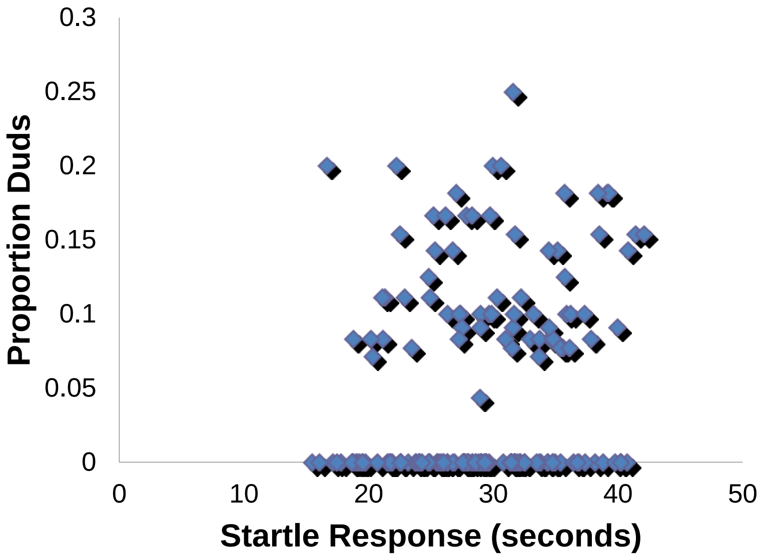

Figure S1. Low correlation between the proportion of ‘dud’ males and a measure of general locomotor activity, startle response. $r^2 = 0.0383$.
