# Supplementary material for: Heritable Variation in Courtship Patterns in Drosophila melanogaster
Source: G3 (Bethesda). 2015 Feb 3;5(4):531–9. doi: 10.1534/g3.114.014811 (PMC4390569; doi:10.1534/g3.114.014811)
Supplement: Supporting Information [file supp_g3.114.014811_TableS1.pdf]

Table S1 Top SNPs from the GWAS for E-A transition probability. Location is noted by both chromosome and genomic location. The minor allele frequency (MAF) was obtained by dividing the number of lines with the frequent allele by the total number of lines tested at that locus. The effect is one-half the difference in mean between lines containing the major and minor allele (Falconer and Mackay, 1996). The *P*-value is from a mixed model analysis that takes *Wolbachia* infection status and relatedness due to shared inversion karyotypes as well as cryptic genetic relatedness into account. Probable gene as well as effect the polymorphism has on that gene is noted as derived from Flybase (Marygold *et al.* 2013). * indicates significant after Bonferroni correction ($\alpha = 0.05$, $P = 2.08 \times 10^{-8}$).

Genomic Location	MAF	Effect	*P*-value	Gene	Site Class
3R _23010872	0.075	-0.0062	6.97E-09*	*Ser*	Intron
*3R*_21296904	0.054	-0.0075	9.89E-09*	*Fur1*	Intron
*3L*_3706152	0.086	-0.0051	1.31E-07	Intergenic	N/A
*3R*_23009915	0.061	-0.0061	2.03E-07	*Ser*	Intron
*X*_20318587	0.129	-0.0044	3.10E-07	*CG42578*	Downstream
*3R*_22864199	0.0556	-0.0065	4.46E-07	*NepYr*	Intron
*3R*_23003275	0.067	-0.0056	5.04E-07	*Ser*	Synonymous
*3R*_22998908	0.073	-0.0056	5.54E-07	*Ser*	Synonymous
*3L*_15807977	0.058	-0.0067	5.92E-07	*fwe*	Intron
*3L*_3706141	0.097	-0.0047	6.65E-07	Intergenic	N/A
*3R*_24359687	0.068	-0.0057	7.41E-07	*Ppn*	Intron
*3R*_23004871	0.100	-0.0047	8.85E-07	*Ser*	Intron
*3R*_22864182	0.061	-0.0060	1.21E-06	*NepYr*	Intron
*3R*_12558223	0.150	-0.0042	1.50E-06	*Ubx*	Intron
*3R*_23006864	0.055	-0.0061	2.03E-06	*Ser*	Intron
*2R*_3790141	0.095	-0.0047	2.42E-06	*CG30377*	Intron
*3R*_23005060	0.067	-0.00523	2.79E-06	*Ser*	Intron
*2R*_9824749	0.200	-0.0035	2.93E-06	*shot*	Intron
*3R*_6210960	0.342	-0.0029	2.93E-06	*mun*	Intron
*X*_800458	0.085	-0.0053	3.12E-06	*e(r)*	Upstream
*3R*_8624797	0.490	-0.0026	3.29E-06	Intergenic	N/A

Genomic Location	MAF	Effect	*P*-value	Gene	Site Class
*3R*_3007818	0.059	-0.0058	3.70E-06	*Ser*	Intron
*3L*_706240	0.072	-0.0048	3.73E-06	Intergenic	N/A
*3L*_6821987	0.094	-0.0048	4.07E-06	*CG13024*	Start gained
*3R*_2999273	0.061	-0.0056	4.53E-06	*Ser*	Synonymous
*3R*_3001738	0.0549	-0.0057	4.86E-06	*Ser*	Intron
*2L*_3477359	0.237	-0.0033	5.49E-06	*CG42784*	Intron
*3L*_3766554	0.198	-0.0036	6.12E-06	*bru-3*	Intron
*3R*_2925879	0.063	-0.0059	6.20E-06	*eater*	Upstream
*3R*_3012273	0.069	-0.0055	6.21E-06	*Ser*	Intron
*2R*_312457	0.385	-0.0025	6.26E-06	*CG42321*	Intron
*3R*_2864293	0.158	-0.0039	7.03E-06	*NepYr*	Intron
*X*_1066955	0.115	-0.0038	7.24E-06	Intergenic	N/A
*2R*_586976	0.106	-0.0041	7.26E-06	*wuc*	Upstream
*3R*_2999769	0.067	-0.0053	7.46E-06	*Ser*	Synonymous
*X*_1067153	0.114	-0.0038	7.65E-06	Intergenic	N/A
*3R*_3002423	0.105	-0.0042	8.10E-06	*Ser*	Intron
*2R*_9337098	0.106	-0.0037	8.37E-06	*cnn*	Intron
*3R*_23009365	0.062	-0.0054	8.74E-06	*Ser*	Intron
*X*_21066980	0.110	-0.0039	8.75E-06	Intergenic	N/A
*3R*_24360681	0.055	-0.0058	8.77E-06	*Ppn*	Intron
*3R*_18106804	0.288	0.0030	8.79E-06	*SKIP*	Intron
*2R*_7000740	0.110	-0.0041	9.08E-06	Intergenic	N/A
*3L*_13938151	0.269	0.0027	9.10E-06	*CG32137*	Intron
*2R*_9312454	0.394	-0.0024	9.31E-06	*CG42321*	Intron
*3L*_13766585	0.195	-0.0035	9.49E-06	*bru-3*	Intron
*2R*_6301231	0.200	-0.0034	9.80E-06	*CG42732*	Intron
*3L*_2799032	0.354	0.0027	9.99E-06	*CG43444*	Intron
